# On the Practical Study of Botany, by Means of Chemical Analysis:—Chiefly Abridged from the Original Papers of Dr. T. S. Hermbstaedt, &c.

**Published:** 1799-06

**Authors:** 


### *On the practical ſtudy of Botany, by means of Chemical Analyſis:—Chiefly abridged from the Original Papers of Dr.* T. S. Hermbstaedt, *&c.*

[Continued from Number III. p. 263 to 269.]

Before we proceed in the chemical analyſis of vegetable bodies, it will be neceſſary to exhibit a table of their various acid and ſaline ingredients.

A. Eſſential acid ſalts.

*a.* The tartaric acid.

*b.* The oxalic acid.

B. Neutral ſalts.

*a.* The ſulphat of potaſs.

*b.* The nitrat of potaſs.

*c.* The muriat of potaſs.

*d.* The tartarite of potaſs.

*e.* The malat of potaſs.

*f.* The oxalite of potaſs.

*g.* The ſulphat of ſoda.

*h.* The muriat of soda.

*i.* The tartarite of volatile alkali, or ammonia.

C. Semi-salts (salia media).

*a.* The malat of lime.

*b.* The tartarite of lime.

*c.* The oxalite of lime.

Besides these different saline substances, there exists in all vegetables an inert matter, possessing no peculiar qualities, and constituting the greatest proportion in almost every vegetable body. This fibrous matter is the residuum of plants, after frequent boiling in water, and after all the other parts have been extracted. It will be readily conjectured, that we here allude to the *vegetable fibre*, consisting of those canals and capillary tubes in which the various constituent parts were preserved previous to their extraction. Upon reducing this fibre by the action of fire, in a pneumato-chemical apparatus, we obtain hydrogen gas (inflammable air), carbonic acid gas (fixed air), and carbonate of lime (carbon), from which last, by means of combustion in atmospheric air, the base of carbon escapes, and calcareous earth alone remains behind.

We have now treated of the different elementary constituents of vegetables, which are essentially distinct from each other, without enumerating the almost endless subdivisions and modifications to which the acids, the essential acid salts, and the neutral and semi-salts, are still subject. The number of them is certainly very considerable; for which reason, greater accuracy of observation, and more indefatigable exertion, will be required in the analytical process, than have hitherto been bestowed upon it. Notwithstanding every attempt to lessen the great number of these constituent parts, we cannot exclude any one without trespassing upon the established laws of nature. But on the other hand, the advantages which a chemical analysis, conducted upon these principles, promises to the enquirer, are so extensive, that they will amply reward the labours which the medical and philosophic chemist may bestow on this fascinating and profitable pursuit.

---

After this necessary digression, we shall return to the subject of experimental analysis, and only observe, with respect to the combination subsisting between the tartaric acid and potash, that this compound is met with in many vegetable bodies, sometimes in a state of saturation, and sometimes in a state where the acid predominates. These salts are chiefly discoverable by the circumstance, that, on red-hot coals, they emit the smell of burnt tartar, and leave behind a residuum consisting of an alkaline carbon.—The oxalic salts may also be readily discovered, by precipitating a solution of them in common springwater; water; and by the acid vapours they emit, if expofed to the action of ignited coals.

The neutral and femi-faline combinations before fpecified, are not unfrequently found as formative or elementary parts in vegetables: their refpective phyfical properties, however, need not be detailed in this place, as the ftudent will readily obtain this part of botanical and medical knowledge from almoft every introductory work on chemiftry and pharmacy. In order to enable the reader to undertake a complete chemical analyfis of vegetables, founded upon general principles, it would be neceffary to give a detailed defcription of the different principles, or elementary conftituents, of vegetable organization, each in their feparate original ftate, affording, at the fame time, an accurate characteriftic of every external, as well as chemical criterion, to prevent any ambiguity or confufion. This the author of the prefent effay could not undertake, in the limited compafs allotted to the inquiry; but he has pledged himfelf to beftow the requifite time and labour on the compofition of a work, which may ferve as an elementary code, exclufively devoted to the fubject of botanical analyfis.

Having premifed thefe obfervations, we refume the practical part of the treatife:—

*Neutral Salts, and Vegetable Acid Salts.*

In examining thefe fubftances, we ought to proceed in different ways, according as we have opportunities of employing frefh or dried vegetable matter. The whole difference, however, merely depends upon this circumftance, that in the former cafe it is not neceffary to make a decoction of the plants, but only to analyfe the newly-expreffed fap: and that if dry fubftances be ufed, a faturated decoction of them fhould be prepared in diftilled water; with which we may perform the analytical procefs in the manner following:

1ft. Let a flip of litmus paper be fufpended in a portion of fuch decoction; and let another portion of it be mixed with the carbonated potafs (in a dry and cryftallized ftate): if, after fome time, the paper become of a red colour, and, upon the addition of an alkaline falt, an effervefcence take place, thefe are undoubted proofs that certain free acids, which are of a vegetable, and not a mineral nature, are contained in the vegetables. In order to inveftigate the properties of thefe vegetable acids, we fhould proceed in the manner following:

2dly. Let a portion of the decoction before mentioned be diluted with diftilled water, and a few drops of the folution of lime in the muriatic acid be added to the mixture. If, after fome hours, or even immediately, a preciprecipitate ſhould be formed, and if the ſubſtance thus precipated ſhould be again ſoluble by adding a few drops of nitrous acid, it is then evident that the plant affords free oxalic acid, from which we may infer the preſence of the eſſential ſalt of ſorrel.

3d. But if, in the preceding experiment, no precipitation ſhould take place, another portion of the decoction, previouſly diluted with a ſmall quantity of the ſolution of lead in the acetic acid, may be employed. Should then a precipitate be formed, which is again ſoluble upon the addition of a ſmall portion of nitric acid, it is manifeſt, that the vegetable under experiment contains not only free tartaric acid, but that it will yield real tartar *.

As, beſides theſe ingredients, there are alſo to be met with, in vegetables, a variety of neutral ſalts, which contain ammonia, or even potaſs, combined with ſulphuric, muriatic, or nitric acid, their exiſtence ought likewiſe to be aſcertained by chemical analyſis.

4thly. In order to diſcover the ammoniacal neutral ſalts, the decoction of vegetable ſubſtances muſt be inſpiſſated to the conſiſtence of a ſyrup, and, while yet warm, mixed with a third part (of its own weight) of the dry cauſtic potaſs. Upon holding a little wooden ſtick, moiſtened in concentrated acetic acid, over this mixture, viſible white vapours will riſe, if it really contain an ammoniacal neutral ſalt; but unleſs that be the caſe, no ſuch vapours will appear.

5thly. The ſulphurated neutral ſalts may be diſcovered by diluting the uſual decoction with diſtilled water, and adding to it a little of the ſolution of barytes in the acetic acid. If it be impregnated with ſulphuric acid, a precipitation will be the conſequence.

6thly. The muriatic neutral ſalt may be diſcovered in a ſimilar manner, by adding to the mixture a few drops of a ſolution of ſilver in the ſulphuric acid, which, if it produce a precipitate, will infallibly ſhew the preſence of muriatic acid.

7thly. The exiſtence of nitre in vegetable bodies may be aſcertained in two different ways: either by evaporating a few ounces of the decoction to dryneſs, and afterwards burning the ſubſtance ſo dried in a ſmall crucible, when

* If by the processes here detailed, a free acid cannot be obtained, either by means of the potass or litmus paper, and the decoction nevertheless affords precipitates with the re-agents before enumerated; and if the matters precipitated agree in their affinity with those already described, we may reasonably conclude, that perfectly neutral salts are present, which have the acids above specified for their bases.

when, if nitre be an ingredient, a flight detonation will fhew its prefence: or by mixing a part of the decoction with the fourth part of a mixture, confifting of equal parts of alcohol and concentrated fulphuric acid; which compound fhould ftand four and twenty hours in a bottle, fecured with a glafs ftopper. Should nitre be contained in it, the mixture, on opening the bottle, will emit the fmell of dulcified nitric acid.

If we wifh to afcertain the exiftence of neutral falts and vegetable acid falts in vegetable fubftances, in a recent ftate, the labours of the inquirer will neither be fo complicated nor fatiguing as in a dry ftate. It requires little more than to reduce the plants, or other vegetable productions, to the confiftence of a pulp, in a ftone mortar, to exprefs the juice by means of proper machines, and, after having allowed it to ftand for fome hours, to pafs it through a filter. Thus prepared, it may be analyfed, firft, by immerfing in it litmus paper, or adding to it the proportion of carbonated potafs before ftated. In order to afcertain the prefence of free acids in general, and to difcover the nature and properties of the particular acids, as well as of the neutral falts contained in the juice of fuch vegetables, the different proceffes already pointed out, may be employed in their fulleft extent.

(To be resumed in our next Number.)

